# Knowledge, Attitude, and Practice in a Sample of the Lebanese Population Regarding Cholera

**DOI:** 10.3390/ijerph192316243

**Published:** 2022-12-04

**Authors:** Diana Malaeb, Malik Sallam, Samar Younes, Nisreen Mourad, Abir Sarray El Dine, Sahar Obeid, Souheil Hallit, Rabih Hallit

**Affiliations:** 1 Department of Pharmacy Practice, College of Pharmacy, Gulf Medical University, Ajman P.O. Box 4184, United Arab Emirates; 2 School of Pharmacy, Lebanese International University, Beirut, Lebanon; 3 Department of Pathology, Microbiology and Forensic Medicine, School of Medicine, The University of Jordan, Amman 11942, Jordan; 4 Department of Clinical Laboratories and Forensic Medicine, Jordan University Hospital, Amman 11942, Jordan; 5 Department of Translational Medicine, Faculty of Medicine, Lund University, 22184 Malmö, Sweden; 6 Department of Biomedical Sciences, School of Pharmacy, Lebanese International University, Bekaa, Lebanon; 7 Pharmaceutical Sciences Department, School of Pharmacy, Lebanese International University, Bekaa, Lebanon; 8 Department of Biomedical Sciences, School of Arts and Sciences, Lebanese International University, Beirut P.O. Box 146404, Lebanon; 9 Department of Social and Education Sciences, School of Arts and Sciences, Lebanese American University, Byblos, Lebanon; 10 School of Medicine and Medical Sciences, Holy Spirit University of Kaslik, Jounieh P.O. Box 446, Lebanon; 11 Research Department, Psychiatric Hospital of the Cross, Jal Eddib, Lebanon; 12 Applied Science Research Center, Applied Science Private University, Amman 11931, Jordan; 13 Department of Infectious Disease, Bellevue Medical Center, Mansourieh, Lebanon; 14 Department of Infectious Disease, Notre Dame des Secours, University Hospital Center, Byblos, Lebanon

**Keywords:** cholera, knowledge, attitude, practice, KAP, Lebanon, outbreak, general population, *Vibrio cholerae*, acute diarrheal illness

## Abstract

The evaluation of knowledge, attitude, and practices towards an emerging disease is an essential component of public health preventive measures during an outbreak. In October 2022, an outbreak of cholera was reported in Lebanon, which is the first to be reported in the Middle Eastern country for 30 years. This study aimed to explore the level of knowledge as well as attitude and practice of the general public in Lebanon towards cholera. A self-administered structured questionnaire was distributed via an online link to individuals living in Lebanon during October–November 2022. The survey instrument comprised items to assess the sociodemographic data; questions on knowledge about cholera symptoms, transmission, and prevention; as well as attitude and practice questions. Our study involved 553 participants, with a median age of 24 years and a majority of females (72.5%). The results showed that the majority of respondents correctly identified diarrhea as a symptom of cholera and recognized the spread via contaminated water and food. Having a university level education compared with secondary school or less (adjusted odds ratio (aOR) = 2.09), being married compared with single (aOR = 1.67), and working in the medical field compared with unemployed (aOR = 4.19) were significantly associated with higher odds of having good cholera knowledge. Having good knowledge compared with having a poor level of cholera knowledge (aOR = 1.83) and older age (aOR = 1.03) were significantly associated with higher odds of having a good attitude towards cholera. The current study showed an overall high knowledge score on cholera among the Lebanese population. Nevertheless, gaps in cholera knowledge were identified and should be addressed, particularly among workers in the medical field. Thus, we recommend targeted health education to the general population that aims to strengthen the health resilience in the community.

## 1. Introduction

Cholera, an acute bacterial infection, is a life-threatening diarrheal disease that is caused by a waterborne pathogen called *Vibrio cholerae* [1,2]. The main clinical characteristic for cholera is watery diarrhea that ranges from mild to moderate symptoms managed with oral rehydration solutions to severe symptomatic cases of profuse watery diarrhea and dehydration, which can lead to death if left untreated [3]. In addition to the watery diarrhea, vomiting and abdominal colic are other clinical manifestations encountered in infected patients [2,3,4].

Depending on the structure of lipopolysaccharides O antigen, *V. cholerae* is classified into serogroups [5]. However, *V. cholerae* is a highly contagious and diverse species with more than 200 serogroups, only two of which (O1 and O139) are toxigenic and thrive in crowded housing conditions with poor sanitation and unhygienic conditions [6]. These two serogroups release cholera toxin, cause clinical manifestations of the disease, and have been linked to the cholera outbreaks globally [6,7].

Cholera is transmitted via ingestion of an infective dose of *V. cholerae* in contaminated water or food linked to poor sanitary measures [8,9]. The diagnosis of cholera through rapid testing is integral as it provides a preliminary result to start a targeted treatment plan [10,11].

A key aspect in cholera outbreak management is the application of a multi-sectoral preventive approach. This approach includes a combination of the following measures: surveillance, sewage systems management, water sanitation, public hygiene, social mobilization, treatment, and oral cholera vaccines, all of which are considered essential to control the outbreak and to reduce mortalities [12,13]. Cholera vaccination can be considered an effective prophylactic measure as it controls and prevents infection, particularly for travelers to an outbreak region [14].

An important contributing factor to the unabated spread of cholera is the inadequate knowledge, attitude, and practices (KAP) among the general public about its possible modes of transmission, early symptoms and identification, and appropriate treatment according to disease severity [15,16,17,18,19,20]. Hence, it is important to conduct KAP studies among the general public to assess the baseline level of cholera knowledge and identify inappropriate practices [21,22,23]. In turn, this can guide the implementation of public health intervention measures, which can aid in the efforts aiming to control the outbreaks [9,23].

There is a general lack of KAP studies on cholera among the Lebanese people. Different studies from variable regions worldwide found gaps in cholera knowledge as well as a suboptimal level of awareness regarding preventive practices among the general public in Kenya, Saudi Arabia, and Yemen [17,18,24,25]. Community knowledge and practice studies during cholera outbreaks assist in identifying areas that can play a critical role in lowering exposure to infectious agents and thus the cholera morbidity and in shaping appropriate public health responses [26].

The global burden of cholera can be viewed in light of the latest estimates, which pointed to 1.3 to 4.0 million cases annually with 21,000–143,000 deaths as a result of the disease [2]. However, the burden of cholera is unequal globally, with the highest toll of cases and fatalities taking place in Africa, Asia, and the Middle East [4,27,28,29]. Lebanon, a Middle Eastern Mediterranean country, recently declared a cholera outbreak on 6 October 2022, the first in the country since 1993, and the disease continues to spread across the country [12].

Different factors could be linked to the cholera outbreak occurrence in Lebanon. As cholera is a sign of countries’ development status, Lebanon had recently encountered the cholera outbreak, which can be linked to the consequences of a humanitarian crisis that resulted in the disruption of water and sanitation systems, besides the displacement of populations to overcrowded camps with inadequate access to clean water and sanitation with subsequent increased risk of cholera transmission. In addition, the Lebanon healthcare system has been hard-hit by a three-year financial crisis and an explosion at the port of Beirut in August 2020 that destroyed essential medical infrastructure in the capital. In this context, responding to a cholera outbreak may overwhelm the already fragile health system in the country. Thus, assessing the level of knowledge, attitude, and practices regarding cholera among the Lebanese population is extremely important as it has a direct impact on the prevention, control, and management of the disease. Therefore, the aim of this study is to examine the knowledge, attitude, and practice of the general public in Lebanon toward cholera infection and its prevention.

## 2. Materials and Methods

### 2.1. Study Design and Participants

This was a cross-sectional prospective study conducted among a random sample of the Lebanese population from different regions in Lebanon including Bekaa, Baalbek/Hermel, Mount Lebanon, Beirut, North/Akkar, and South/Nabatieh. All people residing in Lebanon aged 18 years or older were considered to be eligible for recruitment. Data were collected through an electronic self-administered questionnaire during October 2022–November 2022 using snowball sampling. The questionnaire link was shared through social media outlets including WhatsApp, Facebook, and LinkedIn and was available for the complete duration of the study. A cover letter that explained the study objectives was included at the beginning of the questionnaire and the time for the completion of the questionnaire was about 5–10 min. A pilot study prior to data collection was performed on 10 participants for the purpose of determining the questionnaire’s clarity and consistency and their results were not included in the analysis of this study.

### 2.2. Ethical Aspects

The study was approved by the Psychiatric Hospital of the Cross Ethics Committee (HPC-035-2022). All participants agreed to participate and provided an electronic informed consent. Before proceeding to the first part of the questionnaire, participants had to electronically indicate their informed consent by checking three mandatory options confirming their comprehension of the voluntary and confidential nature of their participation and their agreement to be enrolled in the study.

### 2.3. Study Instrument and Outcomes

The study questionnaire was composed of two parts. The first part included general sociodemographic information of the participants relating to age, sex, area of residence, level of education, occupation, marital status, household crowding index (defined as the number of persons divided by the number of rooms in the house) [30], and the number of children in the house. Financial burden was assessed by one question where the participant was asked to rate it from 1 = lowest financial burden to 10 = extreme financial burden. The second part comprised specific questions on knowledge, attitude, and practices about cholera infection and prevention among the community members. The questionnaire was prepared from previous studies that assessed knowledge, attitude, and practice towards cholera [17,18]. The questionnaire was prepared initially in English and was then translated into Arabic by a translator who is not involved in the study. It included questions to assess knowledge on cause, transmission, treatment, and prevention measures of cholera and another set of questions to assess water sources, water hygiene, and sanitation practices, as well as care-seeking practices for the management of cholera such as type of treatment and site of treatment.

Regarding attitude, it was assessed by including statements concerning the risks of cholera, perceived efficacy of different prevention measures, and by asking about participants’ perception about cholera compared with other causes of diarrhea in terms of severity. One point was given for each correct answer to the knowledge and attitude question. The total score was computed by summing the grades for all questions. The Cronbach’s alpha values in this study were 0.83 for the knowledge scale and 0.71 for the attitude scale.

### 2.4. Sample Size Calculation

The G*Power software was used to determine the sample size. The minimum required sample size was 400 participants, considering an alpha error of 5%, a power of 90%, a minimal model r-square of 5%, and allowing a maximum of 10 predictors to be included in the multivariable model.

### 2.5. Data Analysis

The SPSS software v.25 (IBM Corp., Armonk, NY, USA) was used for the statistical analysis. Cronbach’s alpha was calculated for the knowledge and attitude scores to evaluate reliability. The knowledge and attitude scales were dichotomized into poor and good knowledge/attitude based on >75% correct responses upon assessment of the overall knowledge and attitude scores. The Chi-square test was used to compare two groups, whereas the independent t-test was used to compare two means. Two logistic regressions were conducted taking the dichotomized knowledge and attitude variables as dependent variables; covariates entered in the model were those that showed a *p* < 0.250 in the bivariate analysis. *p* < 0.050 was deemed statistically significant.

## 3. Results

### 3.1. Study Sample Characteristics

A total of 553 participants completed the survey; their mean age was 27.59 ± 10.13 years, while the median age was 24 years (75.2% females). Other sociodemographic characteristics are summarized in Table 1.

### 3.2. Knowledge, Attitude, and Practices of the Lebanese Population towards Cholera

The mean knowledge and attitude scores were 21.73 ± 5.32 and 8.33 ± 2.08, respectively. Moreover, 180 (32.5%) participants had good knowledge, whereas 190 (34.4%) had a good attitude. Finally, 77% of the participants treat or clean the water to make sure you are drinking healthy and safe water, 25.3% on a daily basis, whereas 32.7% get bottled water as their main source of water in the house. The descriptions of the participants’ answers for each knowledge, attitude, and practice question are summarized in Table 2, Table 3 and Table 4, respectively.

### 3.3. Bivariate Analysis

A higher percentage of participants with good knowledge had a university level of education or worked in the medical field. A higher percentage of participants with a good attitude were married, worked in the medical field, and had good knowledge. Finally, a higher mean age was found in the group that had a good attitude compared with the group that had a poor attitude (Table 5).

### 3.4. Multivariable Analysis of Factors Associated with Knowledge and Attitude

Having a university level education compared with secondary school or less (adjusted odds ratio (aOR) = 2.09), being married compared with single (aOR = 1.67), and working in the medical field compared with unemployed (aOR = 4.19) were significantly associated with higher odds of having good knowledge (Table 6, Model 1).

Having good knowledge compared with a poor level of cholera knowledge (aOR = 1.83) and older age (aOR = 1.03) were significantly associated with higher odds of having a good attitude (Table 6, Model 2).

### 3.5. Association between Cholera Knowledge/Attitude and Practice

A significantly higher percentage of participants with good knowledge cleaned the water in the house compared with those with poor knowledge (82.2% vs. 74.5%; *p* = 0.044), whereas having a good attitude was not associated with cholera preventive practice (80.5% vs. 75.2%; *p* = 0.158).

## 4. Discussion

Knowledge and awareness of the general public is as an essential aspect in the management and prevention of cholera outbreaks [20]. Inadequate knowledge and awareness besides improper practices towards the disease can fuel cholera transmission [20]. Cholera outbreaks are linked to poor water, sanitation, and hygiene (WASH) [31,32]. Such circumstances predominate in the regions afflicted by political and economic instabilities including wars with accompanying resurgence of infectious diseases [33]. The current study was conducted amid an ongoing outbreak of this diarrheal disease in Lebanon and Syria, with the latter country suffering from the consequences of civil war since 2011 [34]. The devastating public health impact of war has already been shown with reporting of cholera, typhoid, and hepatitis A outbreaks among Syrian refugees in Lebanon, with increased risk of tuberculosis and leishmaniasis, among other infectious diseases [35,36,37]. Therefore, the increased burden of infectious diseases can weaken an already fragile healthcare system and public health control measures in Lebanon, a country that has already been suffering from political turmoil and economic issues [38].

Cholera outbreaks can be prevented by provision of clean water sources used for drinking and irrigation of agricultural crops [31,39]. Therefore, the emergence of the current cholera outbreak in Syria and Lebanon is fathomable considering the political and economic instabilities in the countries afflicted by cholera [40,41,42]. Specifically, Syria has been struggling with the consequences of the Syrian civil war since 2011, with a subsequent large refugee crisis, besides the negative impact on the health system aggravating its fragility, with limited ability of surveillance and implementation of public health preventive measures [34]. Importantly, the crisis resulted in limited access to clean drinking water in the war zones besides the refugee camps that suffer from poor conditions of living and strains on the availability of clean water and sanitation [43]. Lebanon, a Middle Eastern country that shares the largest borders in the North and Eastern regions with Syria, was one the most neighboring countries to suffer from the burden of the refugee crisis as a result of the Syrian civil war, which further aggravated the political and economic instabilities in the country [44]. Specifically, it is estimated that the number of Syrian refugees welcomed in Lebanon approached 1.5 million in a country with a total population of 6.7 million people as of 2022 [45].

The current outbreak was first reported in Lebanon in early October 2022, in North and Akkar governorates [12,46]. The index case was a Syrian resident in an informal settlement with subsequent report of a nosocomial infection linked to the index case [12].

### 4.1. Cholera Knowledge

In the current study among the general public in Lebanon, a majority of respondents correctly identified diarrhea as a symptom of cholera (94%). In addition, correct knowledge of the spread of *V. cholerae* via contaminated water and food was identified by 97% and 92% of the respondents, respectively. Furthermore, a majority of respondents showed satisfactory/fair levels of cholera knowledge regarding the items tackling the preventive measures. Our results contrast those from several KAP studies in the context of cholera, which showed a generally poor level of knowledge in countries including Bangladesh [22], Saudi Arabia [17], Yemen [24], Democratic Republic of Congo [47], and Tanzania [48]. On the other hand, a qualitative study from South Africa showed better levels of cholera knowledge, likely related to previous reporting of several outbreaks in the country [49]. It is important to consider the timing of our survey in the interpretation of the study results. The current study was based on an electronic survey distributed during October–November 2022, with previous reporting of the cholera outbreak in Syria that grasped media attention [50,51]. In the studies conducted during cholera outbreaks in several countries, a better level of knowledge was reported likely related to the intensified interest in gaining knowledge on matters that affect the daily life of individuals [52]. This highlights the role of policymakers, health professionals, and media in disseminating accurate and timely messages and in building awareness programs that can help in the collaborative efforts aiming to control the spread of this disease [53].

Despite the finding that a majority of respondents in this study have recognized the availability of vaccination, which can help in the prevention of cholera, this result was lower compared with rates of knowledge for other items (64%). Despite the debatable status of the value of cholera vaccination in the control of outbreaks, its value in high-risk individuals traveling to endemic areas is widely accepted [54,55]. In addition, the utility of oral polio vaccines can be seen as a promising tool in the control measures of cholera [56]. Suboptimal levels of knowledge regarding cholera vaccines were not unique to the current study, as they were reported previously in Yemen [24], Bangladesh [22], and in a long-standing refugee camp on the Thailand–Myanmar border [57].

Our study showed suboptimal levels of knowledge regarding cholera vaccines, which can be interpreted by the fact that the cholera vaccination was not available in Lebanon during the current outbreak; this fact triggered the Ministry of Public Health to request oral cholera vaccine doses to cover 400,000 persons from the International Coordinating Group on Vaccine Provision [12]. Directed efforts are needed to increase the approval of the Lebanese population of receiving vaccines to control epidemics [58], including the cholera one. A previous qualitative study from Mozambique displayed hesitancy towards oral cholera vaccination and recommended the promotion of community engagement in the trust building efforts aiming to address cholera epidemic [59]. Another study from Zambia recommended the provision of transparency about cholera vaccine effectiveness and possible side effects to promote vaccine uptake and to tackle the possible conspiratorial beliefs regarding the vaccine [60]. Furthermore, two studies from Tanzania showed the importance of trust in health systems, optimal vaccine campaign implementation, and providing vaccination for free as cholera vaccine acceptance declined if payment was needed as determinants for cholera vaccine uptake [61,62]. This result was also reported in three African regions in the Democratic Republic of the Congo, Kenya, and Tanzania, with a special focus on community interest as a determinant of cholera vaccine acceptance [63].

In addition, the current study showed low levels of knowledge regarding cholera spreading in terms of eating habits and transmission via exposure to animal feces. In this regard, the World Health Organization recommends educating people living in affected regions about safe drinking water, hygiene practices, and food safety as the most effective ways of decreasing the spreading of cholera outbreaks [12]. Awareness campaigns and hygiene promotion are needed to achieve this goal [59,64,65].

In bivariate analysis, better cholera knowledge was linked to female sex and having a university education. Importantly, the occupation in the medical fields was strongly correlated with better cholera knowledge. This result can be linked to previous knowledge acquisition regarding infectious diseases that are part of medical and healthcare curricula besides the potential role of professional training in infectious disease preparedness and response [66]. Despite that, gaps in knowledge among participants working in the medical field should be taken into account as 39% showed poor cholera knowledge and 54% displayed a poor attitude towards the disease. In multivariate analysis, the occupation in the medical field was linked with a four times higher odds of having good cholera knowledge. Additionally, university education and being married were linked to good cholera knowledge in multivariate analysis. A higher educational level was linked to better levels of knowledge regarding various infectious disease including COVID-19 [67]; thus, this result appeared as an expected outcome and might point to the importance of focusing the improvement of cholera knowledge among less educated groups.

### 4.2. Attitude and Practices towards Cholera

Regarding the attitude towards cholera in this study, a majority of respondents showed positive responses manifested by the awareness of the infectious nature of the disease and the potential mortality of this diarrheal infection. In addition, a majority of respondents (89%) displayed the awareness of the association of cholera transmission with drinking water from public places. Similarly, a general positive attitude towards different aspects of cholera outbreaks was reported in various studies worldwide. For example, a study from Bangladesh showed that 97% of the families included had a positive attitude towards cholera and the oral cholera vaccine [22]. Another study from Kenya also showed a similarly positive attitude towards the disease [18].

Regarding the practices, a worrying result in this study is related to the source of drinking water as reported by the participants. The most common source of drinking water was the use of well water, which was reported by 43% of the respondents. The source of drinking water can be linked to various infectious diseases, as it was previously shown that groundwater was associated with possible enteric virus infections [68]. Importantly, a previous study from Uganda showed that contaminated water from an unprotected well was linked to cholera outbreak reported in 2019 in Sembule village [69]. Water source contamination has been reported among the most common risk factors for cholera outbreaks worldwide [70]. Thus, the importance of the quality of drinking water and its disinfection cannot be overlooked in the efforts aiming to control the current cholera outbreak in Lebanon [9]. The current study showed that the practice of drinking water treatment was significantly associated with better cholera knowledge. This result highlights the potentially helpful role of improving cholera knowledge on the practices aiming to prevent such an infection. The promotion of water treatment behavior has been shown to be influenced by factors like perceived risk and self-efficacy besides social norms that should be considered in the intervention strategies aiming to prevent and control the cholera epidemic [71]. The relevance of risk perception in determining water, sanitation, and hygiene behavior has been highlighted in a recent systematic literature review, and this strategy should be considered in intervention measures aiming to improve health behavior in order to control cholera epidemics [72].

Regarding the socio-demographic variables found to be associated with a good attitude towards cholera, older age was linked with a good attitude. An interesting finding in this study was the finding of a better attitude towards cholera among participants with higher knowledge of cholera in multinomial regression analysis. This result highlights the importance of addressing gaps in knowledge regarding cholera, which can subsequently improve the awareness of the disease and might have a positive impact on the efforts aiming to control the current outbreak. In line with our result, an independent correlation between positive attitude towards cholera and high cholera knowledge scores was found in two recent studies conducted in Kenya and Bangladesh [18,22].

### 4.3. Study Strengths and Limitations

The major strength point in this study is its timing, which was concomitant with the ongoing cholera outbreak in Lebanon. Therefore, the current study can be helpful to guide policymakers and health professionals to the gaps in knowledge regarding the infection and to point to areas where improved awareness and better practices can help in cholera outbreak response and control.

On the other hand, the interpretation of the study results should be carried out in light of the following limitations: (1) The study was based on a convenience sample with potential selection bias. The selection bias was manifested in the percentage of female participants that outnumbered males in the study sample and might have influenced the overall cholera knowledge, as females displayed better knowledge. In addition, the study sample, albeit sufficient, might not fully represent the general population in Lebanon as the majority were females, single, unemployed, and with a university level of education. (2) Response bias should be considered for the practices items. (3) The cross-sectional design of the study does not allow the inference of changes in practices and attitude over a longer period, which should be considered in future studies.

## 5. Conclusions

The ongoing cholera outbreak that hit Syria and subsequently Lebanon in early Autumn 2022 requires vigilant actions including raising the level of awareness regarding the disease. The current study showed satisfactory knowledge and a positive attitude towards the disease. However, gaps in knowledge existed that need to be addressed with proper public health messages. The involvement of the stakeholders in the design, content, and implementation of future cholera prevention and control methods can help to raise the community awareness and subsequently reduce the risk from the worrying cholera outbreak. Future educational camping should focus on the improvement in water, sanitation, hygiene, and transmission prevention, including vaccination.

## Figures and Tables

**Table 1:** Sociodemographic characteristics of the participants (*N* = 553).

Variable	N ^1^ (%)
**Sex**, females	416 (75.2%)
**Education**	
Secondary or less	82 (14.8%)
University	471 (85.2%)
**Marital status**	
Single	381 (68.9%)
Married	172 (31.1%)
**Occupation**	
Unemployed	262 (47.4%)
Working in the medical field	122 (22.1%)
Working outside the medical field	169 (30.6%)
**Age categories, years**	
18–24	300 (54.2%)
25–29	86 (15.6%)
30–34	53 (9.6%)
35–39	44 (8.0%)
40–44	26 (4.7%)
45–49	17 (3.1%)
50–54	10 (1.8%)
55–59	11 (2.0%)
60 and above	6 (1.1%)
	**Mean ± SD**
**Age (years)**	27.59 ± 10.13
**Household crowding index (person/room)**	1.10 ± 0.54
**Number of children in the house**	1.11 ± 1.29
**Financial burden**	5.41 ± 2.53

^1^ N: number.

**Table 2:** Description of the cholera knowledge items.

Item	No	I Do Not Know	Yes
	N ^1^ (%)	N (%)	N (%)
**Cholera is a**			
Bacteria	74 (13.4%)	66 (11.9%)	413 (74.7%)
Virus	282 (51.0%)	90 (16.3%)	181 (32.7%)
Fungus	388 (70.2%)	148 (26.8%)	17 (3.1%)
Parasite	385 (69.6%)	146 (26.4%)	22 (4.0%)
**Symptoms of cholera infection**			
Diarrhea	7 (1.3%)	28 (5.1%)	518 (93.7%)
Bloody diarrhea	292 (52.8%)	189 (34.2%)	72 (13.0%)
Vomiting	68 (12.3%)	100 (18.1%)	385 (69.6%)
Fever	95 (17.2%)	88 (15.9%)	370 (66.9%)
Dryness	27 (4.9%)	86 (15.6%)	440 (79.6%)
Loss of appetite	67 (12.1%)	159 (28.8%)	327 (59.1%)
**Ways to prevent cholera infection**			
Cannot be prevented	408 (73.8%)	56 (10.1%)	89 (16.1%)
Herbs	315 (57.0%)	153 (27.7%)	85 (15.4%)
Washing hands	11 (2.0%)	16 (2.9%)	526 (95.1%)
Cooking food well	8 (1.4%)	22 (4.0%)	523 (94.6%)
Heat stored food	170 (30.7%)	109 (19.7%)	274 (49.5%)
Cover food	79 (14.3%)	70 (12.7%)	404 (73.1%)
Boil water	14 (2.5%)	33 (6.0%)	506 (91.5%)
Wash fruits and legumes well	13 (2.4%)	18 (3.3%)	522 (94.4%)
Clean dishes	18 (3.3%)	24 (4.3%)	511 (92.4%)
Use toilets properly	61 (11.0%)	94 (17.0%)	398 (72.0%)
Increase fluid intake	27 (4.9%)	64 (11.6%)	462 (83.5%)
Decrease fluid intake	448 (81.0%)	77 (13.9%)	28 (5.1%)
Increase food intake	197 (35.6%)	169 (30.6%)	187 (33.8%)
Decrease food intake	261 (47.2%)	172 (31.1%)	120 (21.7%)
Oral rehydrating solution (ORS) use	21 (3.8%)	106 (9.2%)	426 (77.0%)
Eat more salt and sugar	127 (23.0%)	197 (35.6%)	229 (41.4%)
Take antibiotics tablets	61 (11.0%)	150 (27.1%)	342 (61.8%)
Intramuscular drugs	180 (32.5%)	208 (37.6%)	165 (29.8%)
Herbal medications	375 (67.8%)	95 (17.2%)	83 (15.0%)
**Spread of cholera**			
Contaminated water	5 (0.9%)	12 (2.2%)	536 (96.9%)
Contaminated food	18 (3.3%)	26 (4.7%)	509 (92.0%)
Bugs	206 (37.3%)	152 (27.5%)	195 (35.3%)
Bad hygiene	16 (2.9%)	22 (4.0%)	515 (93.1%)
Poor sanitation	14 (2.5%)	31 (5.6%)	508 (91.9%)
Kissing	257 (46.5%)	119 (21.5%)	177 (32.0%)
Person-to-person contact	241 (43.6%)	92 (16.6%)	220 (39.8%)
Cholera vaccine exists	66 (11.9%)	131 (23.7%)	356 (64.4%)

^1^ N: number.

**Table 3:** Description of the cholera attitude items.

Item	No N ^1^ (%)	I Do Not Know N (%)	Yes N (%)
**Do you think that:**			
Cholera is contagious	45 (8.1%)	49 (8.9%)	459 (83.0%)
Cholera can cause death	20 (3.6%)	59 (10.7%)	474 (85.7%)
The spread of cholera can be stopped	34 (6.1%)	52 (9.4%)	467 (84.4%)
Travel to infected areas can cause infection	47 (8.5%)	48 (8.7%)	458 (82.8%)
Infection can be caused by eating habits	84 (15.2%)	96 (17.4%)	373 (67.5%)
Cholera infection can be due to drinking water from public places	18 (3.3%)	42 (7.6%)	493 (89.2%)
Can be caused by bad washing hands habits	42 (7.6%)	46 (8.3%)	465 (84.1%)
Can be caused by poor health conditions	14 (2.5%)	40 (7.2%)	499 (90.2%)
Can be caused by the improper use of bathrooms	14 (2.5%)	45 (8.1%)	494 (89.3%)
Can be caused by animal feces	84 (15.2%)	133 (24.1%)	336 (60.8%)
Vaccines can stop cholera pandemic	61 (11.0%)	116 (21.0%)	376 (68.0%)

^1^ N: number.

**Table 4:** Description of the cholera practice items.

Item	No N ^1^ (%)	I Do Not Know N (%)	Yes N (%)
**Place you go if you suspect cholera infection**			
Hospital	78 (14.1%)	30 (5.4%)	445 (80.5%)
Dispensary	179 (32.4%)	45 (8.1%)	329 (59.5%)
Pharmacy	261 (47.2%)	52 (9.4%)	240 (43.4%)
Herbal shop	442 (79.9%)	58 (10.5%)	53 (9.6%)
Family/neighbor	496 (89.7%)	34 (6.1%)	23 (4.2%)
**Treat or clean the water to make sure you are drinking healthy and safe water**			
No	127 (23.0%)		
Yes	426 (77.0%)		
**Frequency of cleaning water tank**			
Daily	140 (25.3%)		
Weekly	83 (15.0%)		
Monthly	106 (19.2%)		
When it gets dirty	224 (40.5%)		
**Source of water in the house**			
Well	236 (42.7%)		
Rainwater harvesting	27 (4.9%)		
Bottled water	181 (32.7%)		
Tank water	87 (15.7%)		
Surface water (river/pool/lake)	22 (4.0%)		

^1^ N: number.

**Table 5:** Bivariate analysis of factors associated with good vs. poor cholera knowledge and attitude.

Variable	Knowledge			Attitude		
	**Poor**	**Good**	***p***	**Poor**	**Good**	***p***
	**(N = 373; 67.5%)**	**(N = 180; 32.5%)**		**(N = 363; 65.6%)**	**(N = 190; 34.4%)**	
Sex			**0.071**			0.988
Males	101 (73.7%)	36 (26.3%)		90 (65.7%)	47 (34.3%)	
Females	272 (65.4%)	144 (34.6%)		273 (65.6%)	143 (34.4%)	
Education			**0.001**			0.192
Secondary or less	68 (82.9%)	14 (17.1%)		59 (72.0%)	23 (28.0%)	
University	305 (64.8%)	166 (35.2%)		304 (64.5%)	167 (35.5%)	
Marital status			0.238			**0.021**
Single	263 (69.0%)	118 (31.0%)		262 (68.8%)	119 (31.2%)	
Married	110 (64.0%)	62 (36.0%)		101 (58.7%)	71 (41.3%)	
Occupation			**<0.001**			**0.009**
Unemployed	193 (73.7%)	69 (26.3%)		178 (67.9%)	84 (32.1%)	
Working in the medical field	47 (38.5%)	75 (61.5%)		66 (54.1%)	56 (45.9%)	
Working outside the medical field	133 (78.7%)	36 (21.3%)		119 (70.4%)	50 (29.6%)	
Knowledge						**<0.001**
Poor				267 (71.6%)	106 (28.4%)	
Good				96 (53.3%)	84 (46.7%)	
Age (years)	27.26 ± 10.77	28.27 ± 8.66	0.274	26.55 ± 9.12	29.58 ± 11.59	**0.002**
Household crowding index (person/room)	1.13 ± 0.55	1.05 ± 0.51	0.116	1.13 ± 0.53	1.06 ± 0.55	0.192
Number of children in the house	1.15 ± 1.33	1.04 ± 1.22	0.367	1.15 ± 1.36	1.03 ± 1.16	0.277
Financial burden	5.36 ± 2.52	5.52 ± 2.54	0.471	5.51 ± 2.51	5.23 ± 2.57	0.216

Numbers in bold indicate significant *p*-values.

**Table 6:** Multivariable analyses.

Multivariable Analyses	*p*	aOR ^1^	95% CI ^2^
**Model 1: logistic regression (using the ENTER method) taking good vs. poor knowledge as the dependent variable (Nagelkerke R^2^ = 16.8%)**			
Sex (females vs. males *)	0.086	1.52	0.94–2.45
Education (university vs. secondary or less *)	**0.026**	2.09	1.09–4.01
Marital status (married vs. single *)	**0.021**	1.67	1.08–2.59
Occupation	**<0.001**		
Unemployed		1	
Working in the medical field	**<0.001**	4.19	2.60–6.75
Not working in the medical field	0.193	0.73	0.45–1.18
Household crowding index	0.805	0.95	0.66–1.38
**Model 2: logistic regression (using the ENTER method) taking good vs. poor attitude as the dependent variable (Nagelkerke R^2^ = 9%)**			
Education (university vs. secondary or less *)	0.102	1.64	0.91–2.96
Marital status (married vs. single *)	0.701	1.10	0.67–1.88
Occupation	0.107		
Unemployed		1	
Working in the medical field	0.280	1.30	0.81–2.11
Not working in the medical field	0.180	0.73	0.47–1.15
Household crowding index	0.596	0.91	0.64–1.30
Knowledge (good vs. poor *)	**0.003**	1.83	1.22–2.72
Age	**0.007**	1.03	1.01–1.06
Financial burden	0.280	0.96	0.89–1.03

* Reference group; numbers in bold indicate significant *p*-values; ^1^ aOR: adjusted odds ratio; ^2^ CI: confidence interval.

## Data Availability

The authors do not have the right to share any data information as per the ethics committee rules and regulations.
